# Pituitary adenomas: a review

**Published:** 2012-11

**Authors:** Mahdi Sharif-Alhoseini, Vafa Rahimi-Movaghar

**Affiliations:** ^*a*^Sina Trauma and Surgery Research Center, Department of Neurosurgery, Tehran University of Medical Sciences, Tehran, Iran.; ^*b*^Research Centre for Neural Repair, University of Tehran, Tehran, Iran.

## Abstract

Pituitary adenomas, as the majority of pituitary neoplasms, are typically benign, slow-growing tumors that arise from cells in the pituitary gland. Those are classified based on secretory products. The functioning (endocrine-active) tumors include almost 70% of pituitary tumors which produce 1 or 2 hormones that are measurable in the serum and cause definite clinical syndromes, that are classified based on their secretory product(s). But non-functioning adenomas are endocrine-inactive tumors. The purpose of this study is to review all types of functioning pituitary adenoma (prolactin, ACTH, GH, TSH, LH and FSH secreting) and non-secreting ones from studies indexed in electronic databases. We describe the symptoms, epidemiology, diagnosis, management, outcome and complications of them separately. Because of physiologic effects of excess hormones, the functioning tumors present earlier than non-functioning adenomas. Therefore patients usually present with symptoms related to a specific hormone imbalance. On the other hand, the mass effect from large pituitary adenomas (often due to non-secreting tumors) may lead to the pressure symptoms, such as headaches, visual field defects (typically loss of peripheral vision first), cranial nerve deficits, hypopituitarism (compression of the normal pituitary gland), pituitary apoplexy (sudden bleeding or outgrowing blood supply), or stalk effect. Apart from prolactinomas, primary treatment for the pituitary adenomas is usually transsphenoidal surgery, and adjunctive treatment may include supervoltage radiation, and occasionally, pharmacologic agents. The most of prolactinomas can be effectively treated with dopaminergic drugs as a primary therapy. Transsphenoidal surgery is an alternative for patients who are intolerant of or resistant to dopamine agonists or when hyperprolactinemia is caused by non-prolactin-secreting tumors compressing the pituitary stalk.

**Keywords:**

Pituitary neoplasm, Prolactinoma, ACTH-Secreting pituitary adenoma, GH-Secreting pituitary adenoma


					Volume 4, Suppl. 1 Nov 2012
Publisher: Kermanshah University of Medical Sciences
URL: http://www.jivresearch.org
Copyright:
Congress of Iranian Neurosurgeons, 14-16 November 2012, Kermanshah, Iran
Paper No. 56
Pituitary adenomas: a review
Mahdi Sharif-Alhoseini a,*
, Vafa Rahimi-Movaghar a,b
a
Sina Trauma and Surgery Research Center, Department of Neurosurgery, Tehran University of Medical Sciences, Tehran, Iran.
b
Research Centre for Neural Repair, University of Tehran, Tehran, Iran.
Abstract:
Pituitary adenomas, as the majority of pituitary neoplasms, are typically benign, slow-growing tumors that arise from
cells in the pituitary gland. Those are classified based on secretory products. The functioning (endocrine-active)
tumors include almost 70% of pituitary tumors which produce 1 or 2 hormones that are measurable in the serum and
cause definite clinical syndromes, that are classified based on their secretory product(s). But non-functioning
adenomas are endocrine-inactive tumors. The purpose of this study is to review all types of functioning pituitary
adenoma (prolactin, ACTH, GH, TSH, LH and FSH secreting) and non-secreting ones from studies indexed in electronic
databases. We describe the symptoms, epidemiology, diagnosis, management, outcome and complications of them
separately. Because of physiologic effects of excess hormones, the functioning tumors present earlier than non-
functioning adenomas. Therefore patients usually present with symptoms related to a specific hormone imbalance. On
the other hand, the mass effect from large pituitary adenomas (often due to non-secreting tumors) may lead to the
pressure symptoms, such as headaches, visual field defects (typically loss of peripheral vision first), cranial nerve
deficits, hypopituitarism (compression of the normal pituitary gland), pituitary apoplexy (sudden bleeding or
outgrowing blood supply), or stalk effect. Apart from prolactinomas, primary treatment for the pituitary adenomas is
usually transsphenoidal surgery, and adjunctive treatment may include supervoltage radiation, and occasionally,
pharmacologic agents. The most of prolactinomas can be effectively treated with dopaminergic drugs as a primary
therapy. Transsphenoidal surgery is an alternative for patients who are intolerant of or resistant to dopamine
agonists or when hyperprolactinemia is caused by non-prolactin-secreting tumors compressing the pituitary stalk.
Key words:
Pituitary neoplasm, Prolactinoma, ACTH-Secreting pituitary adenoma, GH-Secreting pituitary
adenoma
* Corresponding Author at:
Mahdi Sharif-Alhoseini: MD, PhD student of Neurotrauma, Sina Trauma and Surgery Research Center, Department of Neurosurgery, Tehran University
of Medical Sciences, Tehran, Iran. Tel: 09133632609, Email: sharif.mahdi@gmail.com, (Sharif-Alhoseini M.).

				

